# Radioprotective effect of polyvinylpyrrolidone modified selenium nanoparticles and its antioxidation mechanism *in vitro* and *in vivo*

**DOI:** 10.3389/fbioe.2024.1392339

**Published:** 2024-06-19

**Authors:** Wei Li, Xianzhou Lu, Liangjun Jiang, Xiangjiang Wang

**Affiliations:** ^1^ School of Nuclear Science and Technology, Hengyang, China; ^2^ The Affiliated Nanhua Hospital, University of South China, Hengyang, China; ^3^ Hunan Provincial Key Laboratory of Emergency Safety Operation Technology and Equipment for Nuclear Facilities, Hengyang, China

**Keywords:** antioxidation, polyvinylpyrrolidone modification, radiation protection, selenium nanoparticles, nuclear science and technology

## Abstract

**Objective:**

Polyvinylpyrrolidone (PVP) is a commonly used biomedical polymer material with good water solubility, biocompatibility, low immunogenicity, and low toxicity. The aim of this study is to investigate the antioxidant mechanism and clinical potential of PVP modified selenium nanoparticles (PVP-Se NPs) as a new radioprotective agent.

**Methods:**

A laser particle size analyzer and transmission electron microscope were used to characterize PVP-Se nanoparticles prepared by chemical reduction. Human umbilical vein endothelial cells (HUVECs) were used to evaluate the radiation protective effects of PVP-Se NPs. SD rats were employed as an *in vivo* model to identify the most effective concentration of PVP-Se NPs and assess their potential radioprotective properties. Western blot (WB) was used to detect the expression of nuclear factor kappa-B (NF-κB) and mitogen-activated protein kinase (MAPK) signaling proteins in human umbilical vein endothelial cells (HUVECs) and rat liver and kidney tissues.

**Results:**

PVP-Se NPs could reduce the oxidative stress injury and inflammatory response caused by X-ray irradiation in HUVECs and rats, and inhibit cell apoptosis by modulating NF-κB and MAPK signaling pathways. PVP-Se NPs could increase HUVECs viability, reduce apoptosis, inhibit inflammatory factors IL-1β, IL-6 and TNF-α, improve the survival rate of rats, promote antioxidant enzyme activities in cells and rats, reduce malondialdehyde concentration in serum, and reduce the expression of inflammatory factors such as IL-1β, IL-6 and TNF-α in cell supernatant and liver and kidney tissues. PVP-Se NPs could significantly reduce the phosphorylation levels of NF-κB and MAPK pathway-associated proteins in HUVECs and rat liver and kidney tissues (*p* < 0.05).

**Conclusion:**

PVP-Se NPs can protect against radiation-induced oxidative damage by modulating NF-kB and MAPK pathways, providing a theoretical basis and experimental data for their use as an effective radioprotective agent.

## Introduction

Radiation refers to the phenomenon of energy release and propagation (Du et al., 2023). Radiation can be categorized as either ionizing or non-ionizing. Ionizing radiation has greater energy, causing atoms or molecules in a substance to be ionized, leading to DNA damage, gene mutations, cell death, or carcinogenesis (Liu et al., 2023; Ortega et al., 2023). Radiation protection aims to take various measures to reduce or avoid the harm of radiation to human health and the environment. At present, radiation protection consists mainly of three approaches: physical protection, chemical protection, and biological protection (Yang et al., 2020). Among them, chemical protection is the use of specific compounds or elements to enhance organisms’ tolerance to radiation and reduce the harm caused by radiation (Andrady et al., 2023; Javadi et al., 2023).

Selenium (Se) plays a significant role in many physiological functions such as antioxidants, immune regulation, and thyroid hormone synthesis, as described in the report (Li et al., 2021a; Wu et al., 2021; Rao et al., 2022). Research has shown that Se downregulates inflammatory mediators TNF-α, IL-1β and IL-6 gene expressions via TLR2, NF-κB and MAPK signaling pathway in S. aureus-stimulated bMECs, which may be responsible for the anti-inflammatory effect of Se (Wang et al., 2018). As early as 1964, selenium compounds were discovered to have radiation protection effects. It protects organisms from radiation damage through mechanisms such as clearing free radicals, maintaining glutathione levels, and regulating signaling pathways (Shimazu et al., 1964). However, the bioavailability and activity of selenium are influenced by factors such as its chemical form, dosage, and distribution within the organism (Ragini and Arumugam, 2023). For example, In the form of organic selenium compounds, selenium exhibits radiation protection effects only at moderate concentrations, while higher concentrations are toxic to cells (Ragini and Arumugam, 2023). Moreover, selenium compounds demonstrate a combination of preventive (effective before exposure to ionizing radiation) and mitigating (effective after exposure to ionizing radiation, prior to the development of the first clinical signs of radiation sickness), demonstrating their multiple characteristics. Therefore, the development of efficient, safe, and stable selenium-based radiation protection agents is of critical significance.

Nanotechnology refers to the technology of utilizing nanoscale substances or structures to achieve novel functions or performance (El-Morsy et al., 2022). Nanotechnology has a wide range of potential uses in radiation protection due to its unique optical, electronic, magnetic, catalytic, and biological characteristics (Pan et al., 2022). For example, ZnO and TiO_2_ nanoparticles demonstrate effectiveness in shielding against ultraviolet radiation by absorbing and blocking it, thus hindering its penetration into the tissues and cells of human (Cai et al., 2013; Khan et al., 2023). Moreover, another study has shown that CeO_2_ nanoparticles protect gastrointestinal epithelial cells and human lymphocytes from ionizing radiation by reducing oxidative stress and inflammatory reactions (Tarnuzzer et al., 2005; Niu et al., 2007). These findings emphasize the potential of nanoparticles in safeguarding normal cells and tissues from radiation-induced harm. In recent years, nano selenium (Se NPs) has attracted much attention as a new type of selenium-based radiation protection agent. Se NPs exhibit remarkable antioxidant properties, capable of neutralizing free radicals induced by radiation, thereby reducing oxidative stress and protecting cells from damage (Pereira et al., 2022). Moreover, Se NPs has been reported to inhibit radiation-induced cell apoptosis, mitigating direct cellular damage caused by radiation exposure (Gudkov et al., 2023). Furthermore, Se NPs effectively mitigate radiation-induced inflammatory responses, thereby reducing cell death and tissue damage resulting from inflammation (Zahran et al., 2017). However, Se NPs also have some shortcomings, such as easy oxidation, instability, and difficulty controlling particle size (Carroll et al., 2020). Surface modification of Se NPs is therefore one of the most effective ways to enhance their radiation protection properties.

Polyvinylpyrrolidone (PVP) is a commonly used biomedical polymer material with good water solubility, biocompatibility, low immunogenicity, and low toxicity (Ruiz-Fresneda et al., 2023). PVP can be used as an ideal surface modifier to improve the stability, dispersibility, and bioavailability of Se NPs. Previous studies have shown that treating rats with 5 or 10 Gy X-rays and then detecting their cardiopulmonary structure and physiological function can better describe the natural history of this injury (Ghosh et al., 2009). In this study, PVP-Se NPs are prepared by a chemical reduction method and tested *in vitro* and *in vivo* on human umbilical vein endothelial cells (HUVECs) and SD rats, respectively, for radiation protection. Meanwhile, Western blotting was conducted to measure the effects of PVP-Se NPs on HUVECs and MAPK-NF-κB pathway-related proteins in rats’ livers and kidneys, to provide novel preventive measures for radiation protection. These research results will contribute to a deeper understanding of PVP-Se NPs’ radiation protection potential.

## Materials and methods

### Materials and reagents

Chitosan (CTS) and ascorbic acid (Vc) were acquired from Tokyo Chemical Industry in Japan; Human umbilical vein endothelial cells (HUVECs) were obtained from the ATCC cell bank (Cat. NO.AC337632), and cells were cultured in H-DMEM (Hyclone, US) supplemented with 10% fetal bovine serum (Exocell, China), 1% antibiotics (Hyclone, US) at 37°C with 5% CO_2_. Cells were passaged every 3 days or when confluent. Fetal bovine serum (FBS), Dulbecco’s modified Eagle’s medium (DMEM), and SD rats from the Guangdong Provincial Experimental Animal Center were purchased from Gibco Corporation in the United States. CCK8 and Annexin-V/PI kits were acquired from Shanghai Biyuntian Biotechnology Co., Ltd.; CAT(Cat. NO. A007-1, GSH-Px (Cat. NO. A005-1, SOD (Cat. NO. A001-3), MDA (Cat. NO. A003-1) kits were acquired from Nanjing Jiancheng Biotechnology Research Institute; IL-6 (Cat. NO. EK106/2-48), IL-1β (Cat. NO.EK101-48), TNF-α (Cat. NO.EK182-48) ELISA kits were purchased from Lianchuan Biotechnology Co., Ltd.; P-IκB (Catalog: AF2002), IκB (Catalog: AF5002), NF-κB (Catalog: AF0874), p-p65 (Catalog: AF3389), p65 (Catalog: AF5006), p-JNK (Catalog: AF3318), JNK (Catalog: AF6318), p-ERK (Catalog: AF1015), ERK (Catalog: AF0155), p-p38 (Catalog: AF6455), and p38 (Catalog: AF6456) antibodies were acquired from Affinity Corporation. Secondary antibodies used in Western blot analysis were acquired from Abcam.

## Methods

### Preparation and characterization of PVP-Se NPs

Dissolve 0.5 g of CTS and 1.6 g of ascorbic acid (Vc) in 100 mL of 1% (w/w) acetic acid. Incorporate 10 mL of sodium selenite solution containing 0.4 g/10 mL slowly, stirring at 800 rpm/min for at least 8 h at room temperature. Mix the colloid with acetone solution, centrifuge at 12,000 rpm/min for 5 min, and discard the supernatant. Then put the nanomaterials into a dialysis bag, add deionized water for dialysis for 24 h, change the water every 1 h within the first 6 h, and change the water at 12 and 18 h respectively. Remove the nanomaterials, centrifuge at 12,000 rpm/min for 5 min, and remove the supernatant. Vacuum dry and weigh at 50°C to obtain nano-selenium samples. We examined the particle size, morphology, and distribution of PVP-Se NPs utilizing a laser particle size analyzer (DLS) and transmission electron microscopy (TEM).

### *In vitro* biological evaluation of PVP-Se NPs

#### Screening of the optimal concentration and X-ray dose of PVP-Se NPs

Inoculate HUVECs onto a 96-well plate and divide them into a control and experimental group. For the control group, cells were treated with 0, 18, 36, and 72 μg/mL PVP-Se NPs respectively for 24 h, followed by no X-ray treatment. The experimental group was split into eight subgroups: Cells were treated with 0, 18, 36, and 72 μg/mL PVP-Se NPs for 24 h and then subjected to radiation treatment using X-ray doses of 5 Gy and 10 Gy, respectively. Then add 10 μL CCK-8 reaction solution to each hole. Incubate for 2 h, and measure the absorbance at 450 nm using an enzyme-linked immunosorbent assay.

#### Flow cytometry

Flow cytometry (ThermoFisher Attune NxT, United States) is used to detect cell apoptosis. Set up a control group and an experimental group according to the optimal concentration and X-ray dose selected above. Inoculated HUVEC cells were cultured overnight in a 6-well plate. As a control group, PVP-Se NPs were administered at a concentration of 0 g/mL, and as an experimental group, PVP-Se NPs were utilized. After 24 h, all cells were subjected to X-ray irradiation. Each group’s cells were collected and washed twice with PBS after 48 h. Apoptosis was detected immediately after 15 min of double staining with Annexin V-FITC and PI.

#### Detection of cellular oxidative stress levels

The methodology employed for the grouping treatment followed the same protocol as described in “*Detection of cell apoptosis level*”. Subsequently, the concentrations of catalase (CAT), glutathione peroxidase (GSH-Px), superoxide dismutase (SOD), and malondialdehyde (MDA) in the cellular supernatant were quantified using a specialized reagent kit.

#### Detection of cellular inflammatory factor content

This treatment is the same as “*Detection of cell apoptosis level*,” using an ELISA kit and following the instructions to determine the absorbance (OD) value at 450 nm using an enzyme-linked immunosorbent assay (ELISA). In the end, the levels of interleukin-1 β (IL-1β), tumor necrosis factor- α (TNF-α) and interleukin-6 (IL-6) in the supernatant of each cell group were determined according to a standard curve.

#### Western blot analysis

Proteins from each group of cells, the liver and kidney tissues of rats are extracted and quantified using the BCA method. They are separated by SDS-PAGE electrophoresis, and membranes are transferred. After blocking for hours, they were incubated with the primary antibody at 4°C overnight and the secondary antibody at room temperature for 1 h. Finally, chemiluminescence development was carried out.

### *In vivo* efficacy evaluation of PVP-Se NPs

#### Screening of the optimal concentration of PVP-Se NPs for radiation protection in rats

A total of 32 6–8 weeks SD rats were randomly divided into four groups, each containing eight rats. Whole-body 10 Gy (2 Gy/min) X-ray irradiation was performed following intraperitoneal injection of 200 μL of PVP-Se NPs at concentrations of 0, 18, 36, and 72 μg/mL. Once the rats had been irradiated, they were placed in a clean box and returned to the barrier laboratory for cage feeding. Throughout the 15-day period, the number of survivors was counted regularly, and the survival rate was calculated. The optimal concentration of PVP-Se NPs was selected. This study is approved by the Ethics Committee of the Affiliated Nanhua Hospital, University of South China (NO. 2022-ky-21). All animal studies were conducted in accordance with animal care standards, and all experiments were conducted in accordance with the guidelines of the institutional animal ethics committee.

#### HE staining and IHC

At the optimal concentration selected in “*In vivo* efficacy evaluation of PVP-Se NPs,” we set up a control group and an experimental group respectively. On the 15th day after radiation, the rats were euthanized by the cervical dislocation method, and the liver and kidney tissues were quickly removed. They were fixed in 4% formaldehyde for 24 h, and paraffin sections were routinely prepared. An optical microscope was used to observe and photograph them following HE staining.

For IHC, sections of the liver and kidney tissues were treated with xylene and graded alcohol and then subjected to antigen retrieval in 0.01 M citrate buffer. Hydrogen peroxide was used for blockage. The sections were incubated with goat serum for 30 min and then with anti-IL-1β, anti-IL-6 and anti-TNF-α antibodies overnight at 4°C. Subsequently, slides were incubated with biotin-linked secondary antibody and peroxidase-labelled streptavidin followed by a diaminobenzidine (substrate of peroxidase) revelation and counterstaining with Mayer’s hematoxylin. Slices were analyzed under a microscope.

#### Detection of oxidative stress levels

Grouping processing is the same as “*HE staining and IHC*”. The control group received intraperitoneal injection of 200 µL PVP-Se NPs without X-ray irradiation; The experimental group was intraperitoneally injected with 200 µL of PVP-Se NPs and subjected to whole-body 10 Gy (2 Gy/min) X-ray irradiation. Collect serum from rats for 15 days and detect the expression level of oxidative stress.

#### TUNEL staining

The grouping treatment is the same as that described in “*HE staining*.” TUNEL staining was performed after 15 days in order to identify inflammatory factors in the liver and kidneys of the rats. The stained slices were microscopically magnified (×400, Axiovert 25C; Carl Zeiss, Germany) and colored photomicrographs were taken using a digital camera (Canon Eos 1000D, Japan). Pictures were standardized (“autocontrast” function, Adobe Photoshop CS5 Extended, United States) and the number of TUNEL-positive cells was counted in particular grids within VCN (0.22 mm × 0.17 mm), the fusiform layer of DCN (0.16 mm × 0.1 mm), and the ICC (0.45 mm × 0.33 mm).

### Statistical analysis

Statistical analysis was performed using SPSS 22.0, and measurement was defined as mean ± SD. The groups were compared by using one-way ANOVA and Dunnett’s test. Statistics are significant when they have *p* < 0.05.

## Results

### Physicochemical properties of PVP-Se NPs

**FIGURE 1:**
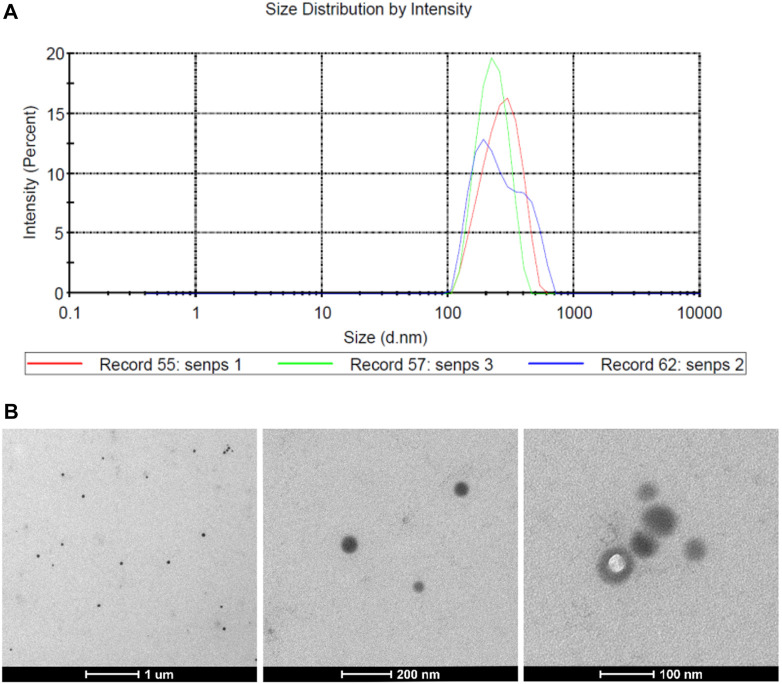
Physicochemical properties of PVP Se NPs **(A)**. The particle size distribution of PVP Se NPs; **(B)**. Transmission Electron Microscopic Observation of PVP-Se NPs.

The average particle size of PVP-Se NPs observed by a laser particle size analyzer was 278.4 nm (Figure 1A). Through transmission electron microscopy, it was observed that PVP-Se NPs were hemispherical in shape, with a smooth surface and no obvious aggregation phenomenon (Figure 1B).

### Effects of PVP-Se NPs and X-rays on the viability of HUVECs cells

**FIGURE 2:**
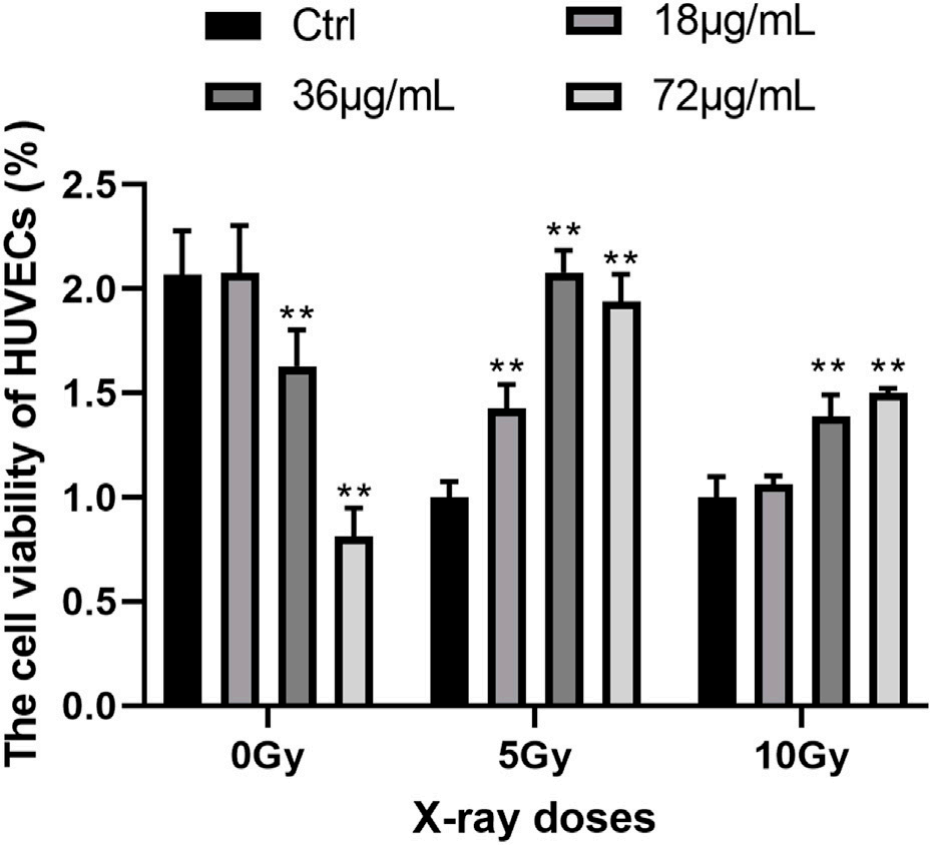
Effects of different concentrations of PVP-Se NPs and doses of X-rays on the viability of HUVECs cells Data are expressed as mean +standard deviation (*n* = 6), **p < 0.01.

To determine the optimal level of PVP-Se NPs and the optimal dose of X-rays, the CCK-8 experiment was conducted. The results showed (Figure 2) that in the absence of radiation treatment, the activity of HUVECs cells decreased as the concentration of PVP-Se NPs increased; After radiation treatment, PVP-Se NPs can significantly increase the cell viability of HUVECs, and with the increase of PVP-Se NPs concentration, the cell viability increases (*p* < 0.01). At two X-ray doses of 5 Gy and 10 Gy, 36 μg/mL PVP-Se NPs showed the best protective effect, and at the same concentration, the cell activity of 10 Gy was higher than that of 5 Gy. Therefore, choose 36 μg/mL concentration and 10 Gy dose for subsequent experiments.

### PVP-Se NPs resist HUVECs cell apoptosis *in vitro*

**FIGURE 3:**
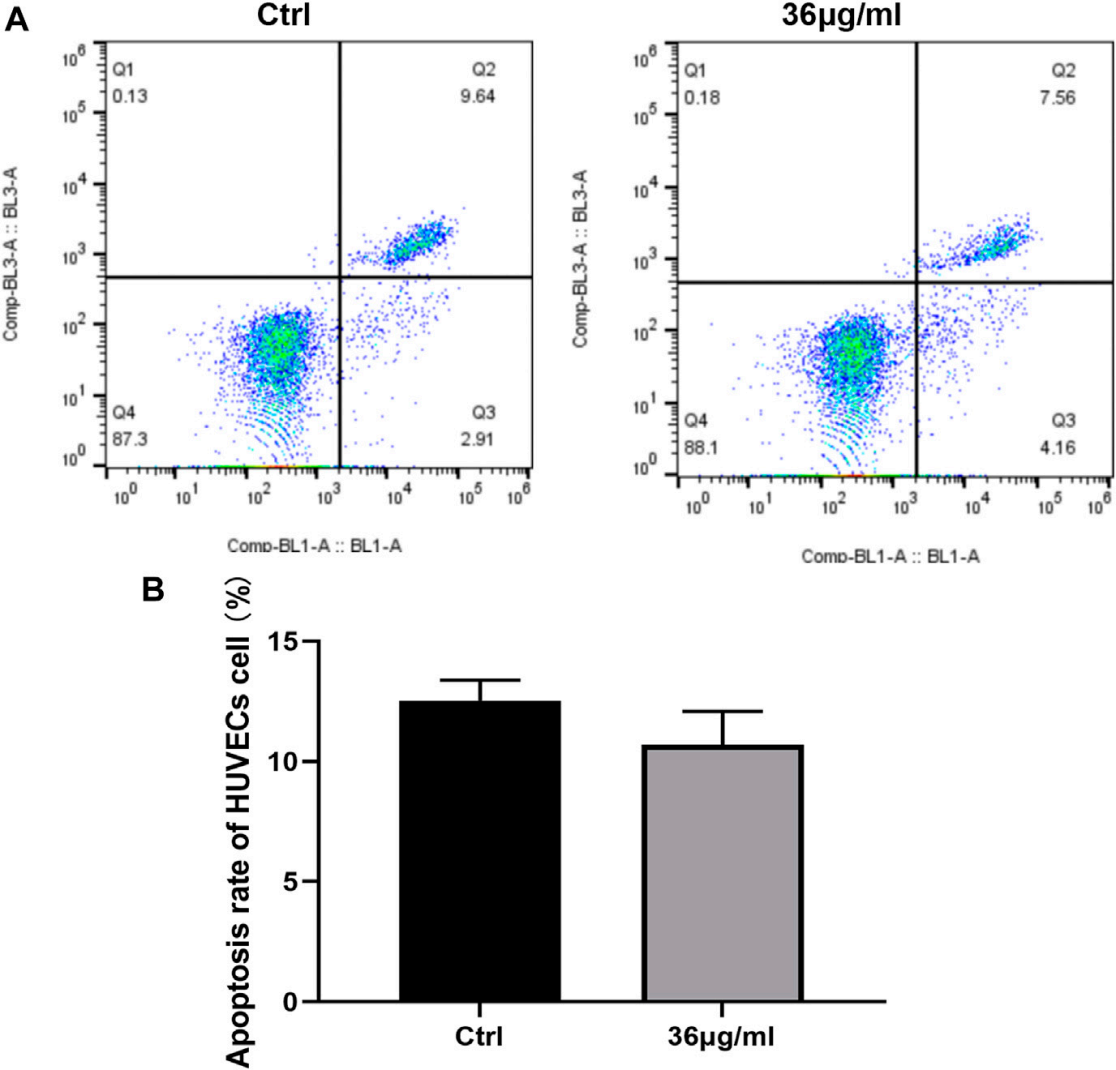
PVP Se NPs inhibit HUVECs cell apoptosis **(A)**. The apoptosis rate was detected using Annexin V-FITC/PI double staining method; **(B)**. Statistical analysis of cell apoptosis rate.

It was found that treated cells with 36 g/mL PVP-Se NPs displayed a lower apoptosis rate than control cells; however, the difference was not statistically significant (Figure 3).

### PVP Se NPs inhibit oxidative stress damage and inflammatory factor levels in HUVECs cells

**FIGURE 4:**
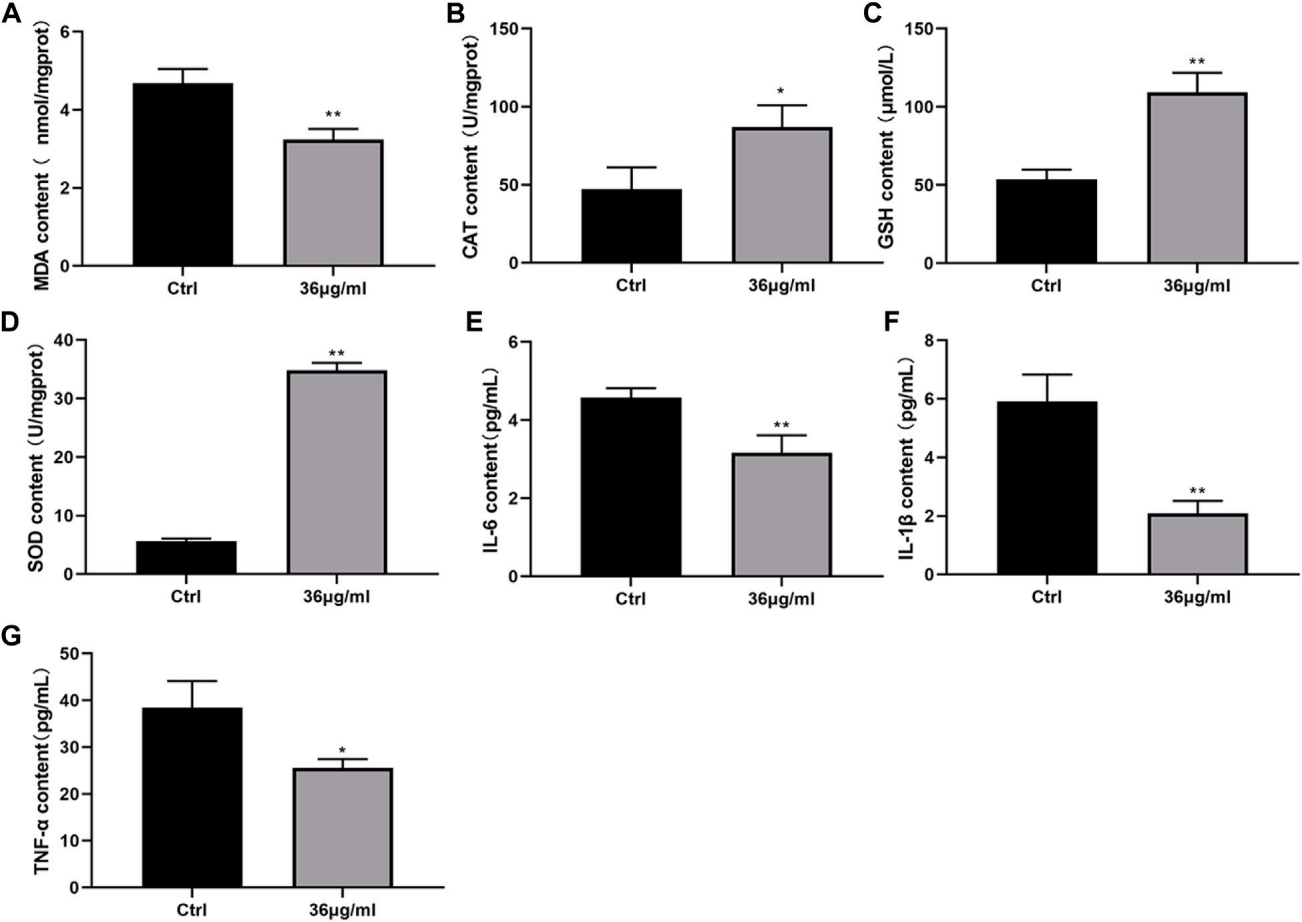
Effect of PVP-Se NPs on oxidative stress damage and inflammatory factor secretion in HUVECs cells **(A)**. MDA content; **(B)** CAT content; **(C)**. GSH-Px content; **(D)**. SOD content; **(E)**. IL-6 content; **(F)**. IL-1β content; **(G)**. TNF- α content. The data is represented as mean ± standard deviation (*n* = 3), *p < 0.05, **p < 0.01.

The levels of oxidative stress factors and inflammatory factors were assessed using an ELISA kit. The findings depicted in Figure 4 indicated that the PVP-Se NPs group exhibited a notable decrease in MDA, IL-6, IL-1β, and TNF-α content, when compared to the control group. Conversely, the levels of CAT, GSH-Px, and SOD were significantly elevated in the PVP-Se NPs group (*p* < 0.05). This indicates that PVP Se NPs can alleviate radiation-induced oxidative stress and inflammation.

### PVP-Se NPs inhibit the expression of HUVECs cell related proteins

**FIGURE 5:**
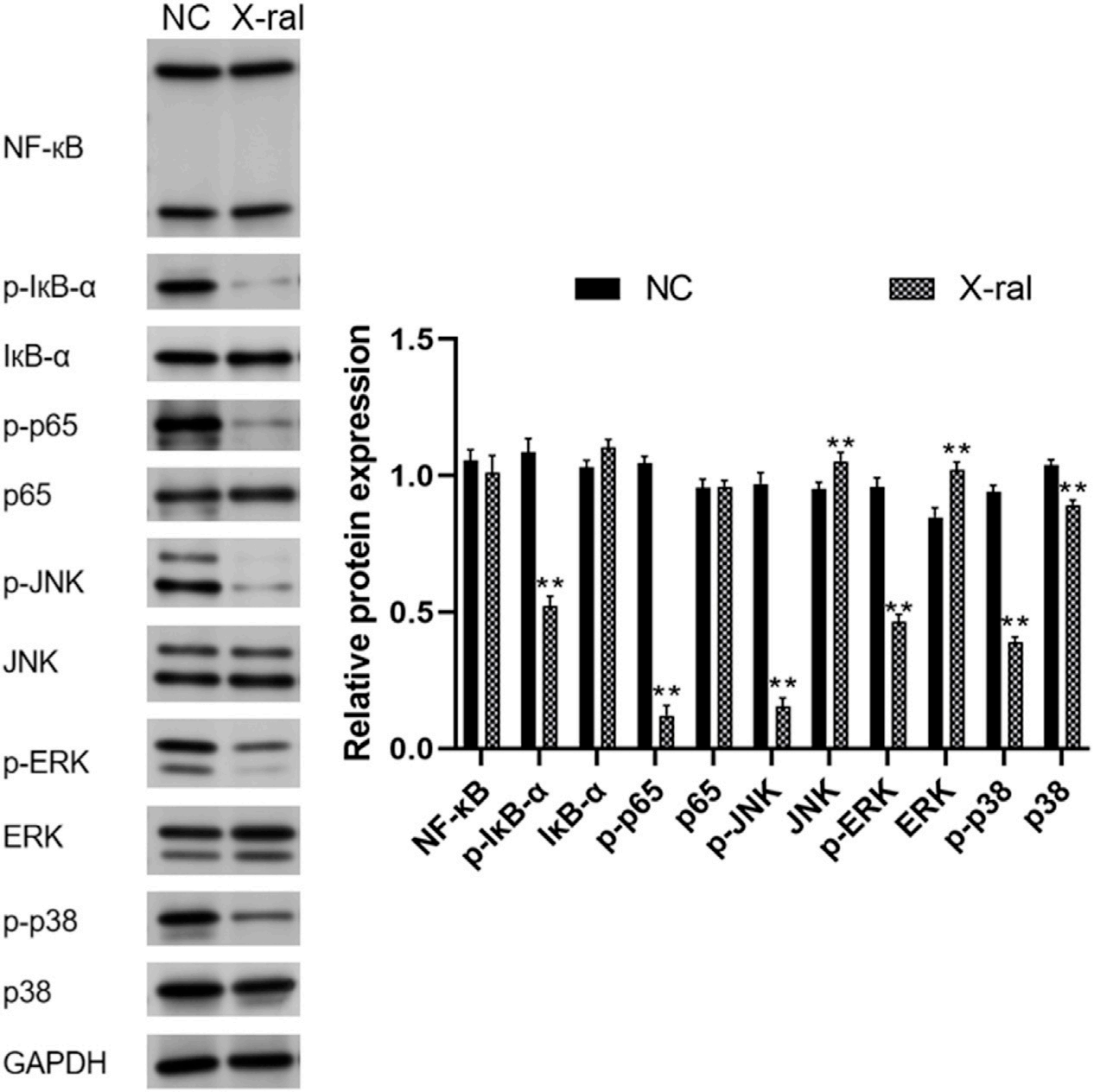
Western blot was performed to detect NF-κB and MAPK-associated proteins in HUVECs.

The expression levels of proteins related to the NF-κB and MAPK signaling pathways were determined by Western blot analysis. The results indicated that in comparison with the control group, PVP Se NPs significantly inhibited the activity of p-IκB-α, p-p65 in the NF-κB pathway, and inhibited the activity of p-JNK, p-ERK, and p-p38 in the MAPK signaling pathway (*p* < 0.05), thereby exerting a radiation protective effect (Figure 5).

### Screening of the optimal concentration of PVP-Se NPs *in vivo* and improvement of radiation damage in rats.

**FIGURE 6:**
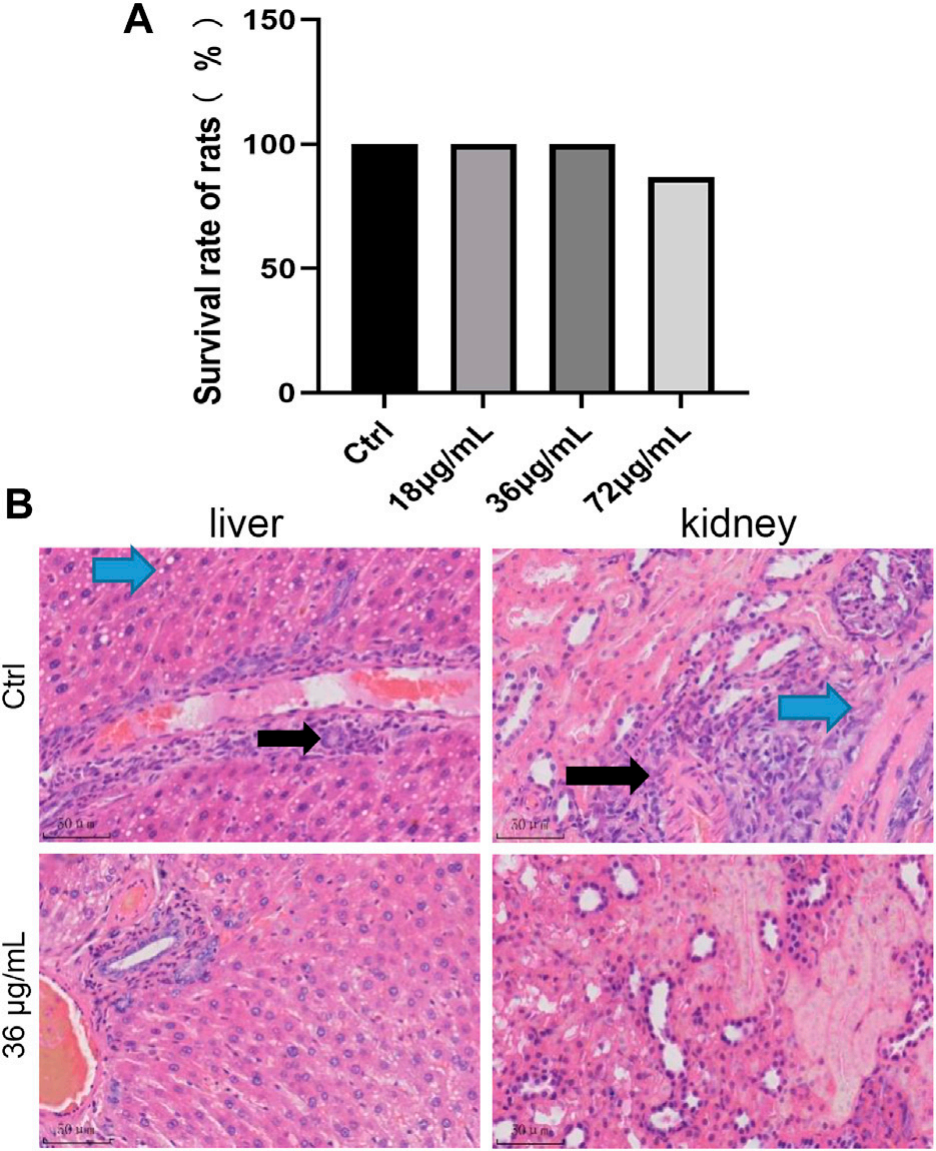
Screening of the optimal concentration of PVP-Se NPs for radiation protection in rats **(A)** 15 days survival rate of rats; **(B)**. HE staining observation of pathological changes in liver and kidney tissues (400 ×).

Without radiation treatment, 0, 18, and 36 μg/mL of PVP Se NPs did not significantly affect the survival rate of rats, 72 μg/mL of PVP Se NPs inhibits rat survival, indicating 36 μg/mL PVP Se NPs is relatively safe for rats, so this concentration was selected for subsequent experiments (Figure 6A).

HE staining shows (Figure 6B) diffuse mild vacuolar degeneration of liver cells in the control group (blue arrow). Mild mononuclear inflammatory cell infiltration can be seen around the central vein and portal vessels (black arrow). No vascular or bile duct hyperplasia was observed in the portal area; In the PVP-Se NPs treated group, the liver cell cords were arranged neatly, and the hepatic sinuses were clearly visible. Interlobular arteries, veins, and interlobular bile ducts in the portal area do not exhibit dilation or proliferation; no inflammatory cells are infiltrated, and fibrous tissue does not proliferate. The control group showed multifocal mild mononuclear inflammatory cell infiltration in the urinary tubulointerstitium (black arrow). Mild fibrous tissue hyperplasia around blood vessels (blue arrow); In the PVP-Se NPs treated group, there was no edema or degeneration of renal tubular epithelial cells, and no exudate or tubular type was found in the lumen. Interstitial blood vessels in the kidney did not dilate or become inflamed with inflammatory cells.

### PVP-Se NPs improve radiation-induced oxidative stress damage in rats

**FIGURE 7:**
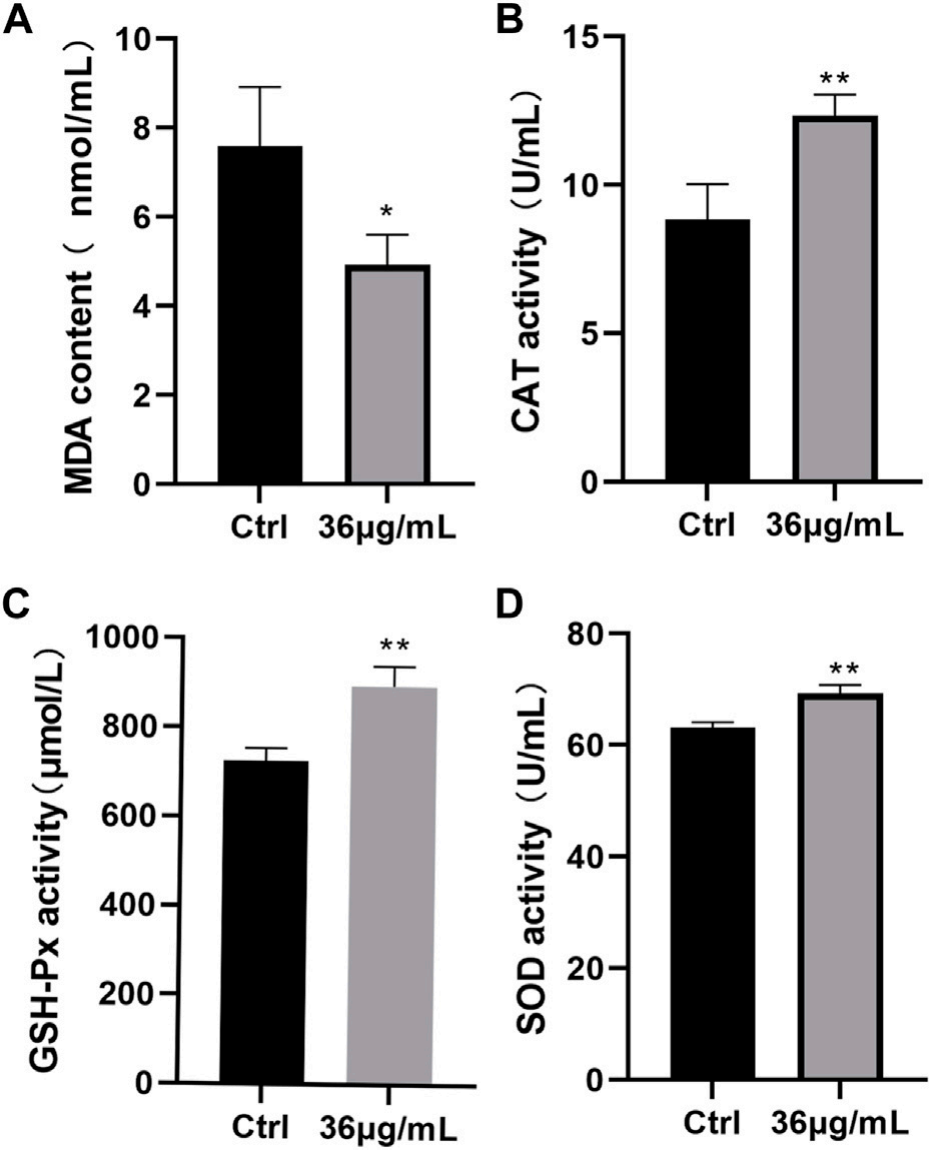
Antioxidant stress effect of PVP-Se NPs in rats **(A)**. MDA content; **(B)**. CAT activity; **(C)**. GSH-Px activity; **(D)**. SOD activity. Mean and standard deviation are used to represent the data (*n* = 4), *p < 0.05, **p < 0.01.

Based on the study results (Figure 7), the PVP-Se NPs group’s MDA content was significantly lower than the control group’s (*p* < 0.05), whereas levels of CAT, GSH-Px, and SOD were significantly higher (*p* < 0.01). This indicates that PVP Se NPs can reduce radiation-induced oxidative stress.

### PVP-Se NPs inhibit radiation-induced cell apoptosis in rats

**FIGURE 8:**
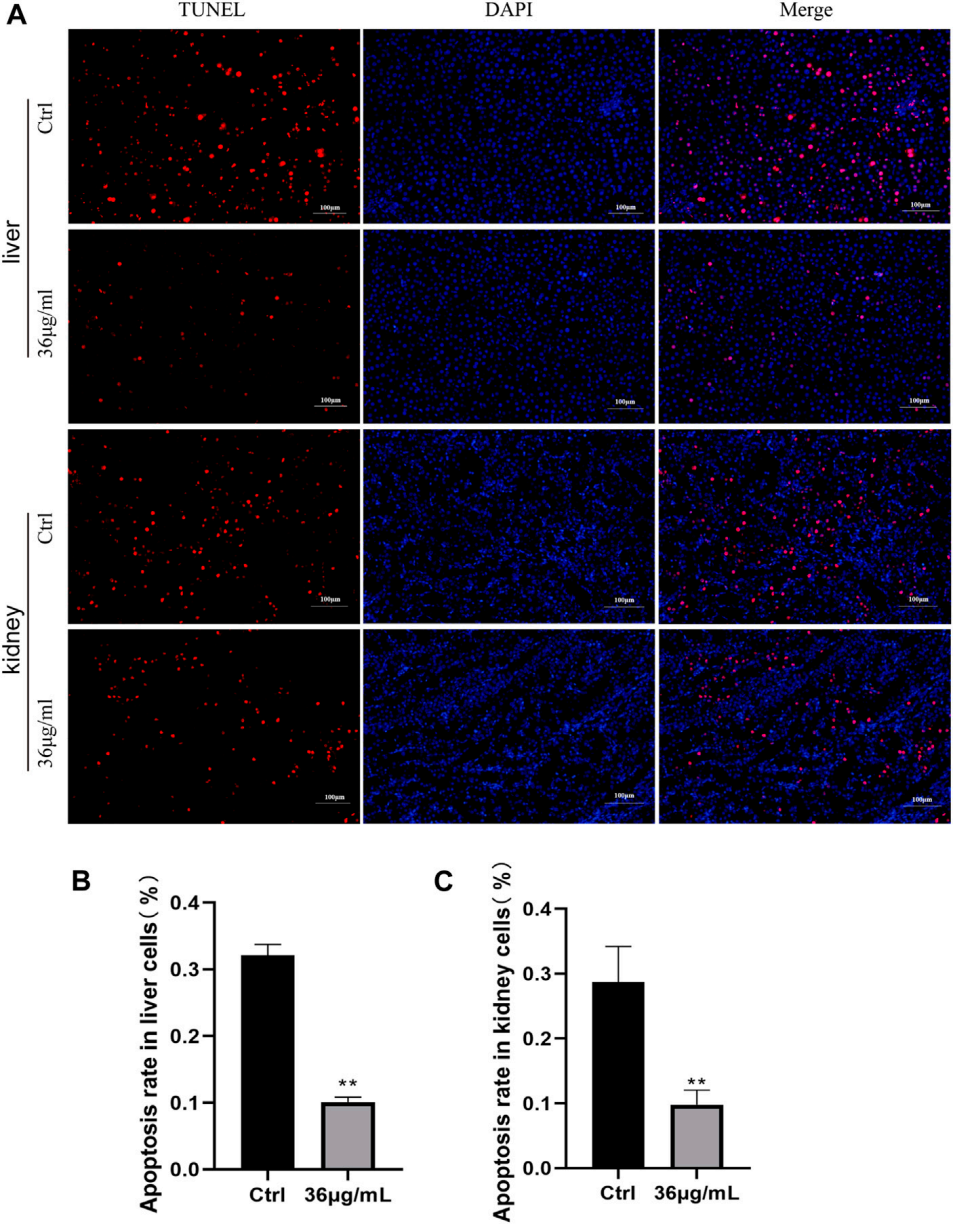
Inhibition of cell apoptosis by PVP Se NPs in rats’ liver and kidney tissues **(A)**. TUNEL staining observation of cell apoptosis in rats’ liver and kidney (400 ×); **(B)**. Statistical analysis of the apoptosis rate in rat liver tissue cells; **(C)**. Analysis of the apoptosis rate in rat kidney tissue cells. Data are presented as mean and standard deviation (*n* = 3), *p* < 0.05, *p < 0.01.

The TUNEL staining results (Figure 8) indicated that compared to the control group, cells treated with PVP-Se NPs had a lower apoptosis rate, indicating that PVP-Se NPs significantly inhibited radiation-induced apoptosis in rats’ liver and kidney tissues (*p* < 0.01).

### PVP-Se NPs inhibit radiation induced inflammatory factor expression in rats

**FIGURE 9:**
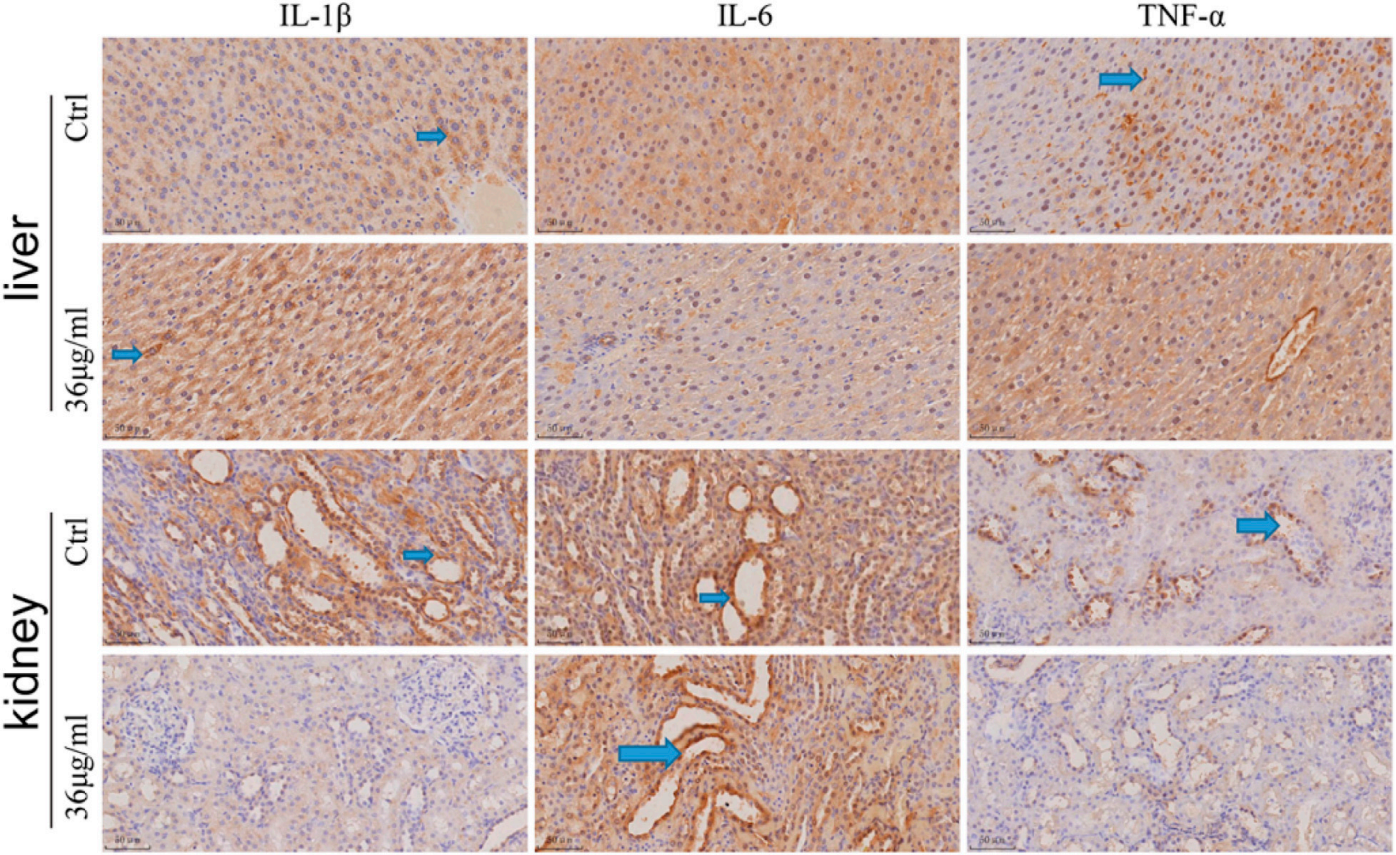
Immunohistochemical detection of inflammatory factors in the rat liver and kidney (40×). The blue arrow shows a positive staining of brownish yellow.

The expression of inflammatory factor IL-1β, IL-6 and TNF-α in rats’ liver and kidney tissues was determined by immunohistochemistry, as illustrated in the figure (Figure 9). Both the control group and PVP-Se NPs group showed positive expression of diffuse hepatocyte cytoplasmic IL-1β, but the positive area of liver cells in the control group was slightly higher than that in the PVP-Se NPs group. The majority of renal collecting ducts and some distal convoluted tubules in the kidney control group and PVP-Se NPs group showed IL-1β positivity, but the levels of positivity in the control group were higher than those in the PVP-Se NPs group. No IL-1β positive reaction was observed in the liver of the control group and PVP-Se NPs group; All kidneys showed IL-6 positive reaction in the collecting duct, and there was no difference in the positive area and staining degree. One case of Kupffer cell TNF-α positive was found in the liver control group, while no positive reaction was found in all other liver samples; One case in the PVP-Se NPs group showed no TNF-α positive reaction in the kidneys, while all other submitted kidneys revealed TNF-α positive reaction in the collecting duct. There was no difference in the positive area and staining degree.

### PVP-Se NPs inhibit the expression of oxidative related proteins in rats

**FIGURE 10:**
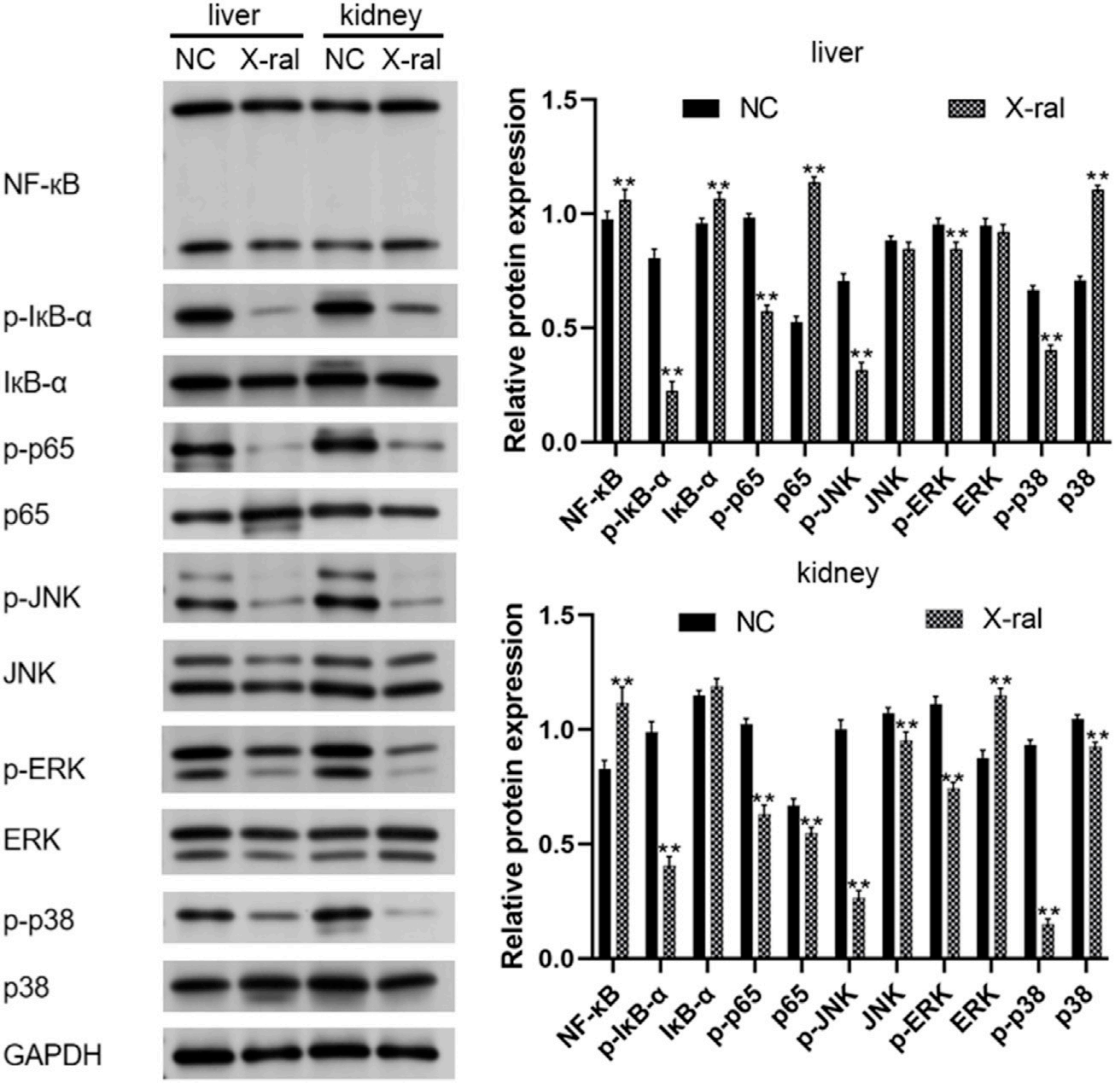
Western blot was used to detect NF-κB and MAPK pathway-related proteins in rats’ liver and kidney tissues.

Western blots were used to analyze NF-κB and MAPK pathway-related proteins in rat liver and kidney tissues. It was found that compared to the control group, PVP-Se NPs significantly inhibited p-IκB-α and p-p65 in the NF-κB signaling pathway (*p* < 0.01). The presence of p-p65 significantly inhibits the expression of p-JNK, p-ERK, and p-p38 in the MAPK regulatory pathway (*p* < 0.05), thereby exerting antioxidant and radiation protective effects (Figure 10).

## Discussion

Finding substances to prevent radiation damage remains one of the urgent focuses in radiation biology. Although the radiation resistance effects of a large number of compounds have been studied (Aygun et al., 2020; Kreusch and Duarte, 2021; Wang et al., 2023), Existing radiation protection methods still fail to fully meet the requirements, especially in terms of key characteristics such as efficiency, action time, flexibility, and usability (Kapustka, 2022). Due to selenium’s antioxidant properties, there is growing research focusing on its utilization in radiation protection. There are many similarities between selenium’s chemical properties and those of sulfur. Nevertheless, some selenium derivatives exhibit a significantly higher ability to scavenge free radicals and peroxides than similar sulfur compounds. Numerous studies have demonstrated Selenium’s antioxidant and protective properties. Selenium compounds protect biological molecules from oxidative stress (Li et al., 2023; Mamgain et al., 2023). However, conventional Se supplements present a range of challenges, including low absorption efficiency and elevated toxicity levels. Therefore, developing innovative systems as carriers of selenium compounds to improve selenium bioavailability and achieve controlled release *in vivo* is of significant significance.

Recent research has revealed that nano-modified inorganic compounds display antioxidant properties (Krishnan Nair et al., 2023). When utilized as therapeutic agents within the body, Se NPs can gradually release biologically available selenium in a controlled manner (Zhang et al., 2001). Compared to molecular forms of selenium, the toxicity of Se NPs is reduced by 50–100 times (Gao et al., 2023). In this study, we synthesized polyvinylpyrrolidone-modified selenium nanoparticles (PVP Se NPs) and explored their protective effects against radiation, along with their potential molecular mechanism. Previous research has suggested that even minimal concentrations of molecular selenium could pose damage to cells (Maynar et al., 2018). Hence, initial screening for the optimal PVP-Se NPs concentration becomes necessary. Experimental findings reveal that the optimal concentration of PVP-Se NPs for both *in vitro* and *in vivo* is 36 μg/mL.

It has reported that ionizing radiation can interact with cellular molecules, triggering an overproduction of free radicals and reactive oxygen species such as hydroxyl radicals, hydrogen peroxide, and superoxide radicals. This cascade of free radicals and reactive oxygen species induces oxidative stress (Shirazi et al., 2011). Oxidative stress damages biomacromolecules, disrupting cellular redox balance and impairing normal cellular function and homeostasis, ultimately culminating in cell death (Clavo et al., 2019). Additionally, ionizing radiation also has the ability to prompt the excessive release of inflammatory factors and activation of inflammatory signaling pathways (Baselet et al., 2019; Wijerathne et al., 2021). In this study, we found that PVP Se NPs significantly reduced the generation of MDA, IL-1, IL-6 and TNF-α increased the generation of GSH, SOD, CAT, and effectively alleviated cell apoptosis in HUVECs and rats. These findings collectively suggest that PVP Se NPs mitigate radiation-induced damage both *in vitro* and *in vivo* by alleviating oxidative stress, inflammatory responses, and cell apoptosis.

To further investigate the potential molecular mechanisms underlying the mitigation of radiation damage by PVP-Se NPs, we utilized Western blot analysis to assess the expression of proteins associated with the NF-κB and MAPK signaling pathways in HUVECs and rats. NF-κB serves as a pivotal nuclear transcription factor implicated in regulating various cellular processes, including proliferation, immunity, and inflammatory responses (Thakur and Ray, 2017). A prior study demonstrated that curcumin mitigates oxidative stress and inflammatory responses in rats by suppressing NF-κB signaling pathway activation, thus ameliorating radiation-induced liver damage (Li et al., 2021b). MAPK functions as a serine/threonine-specific protein kinase involved in regulating essential physiological and pathological cellular processes, including growth, differentiation, apoptosis, and inflammatory responses (Fecher et al., 2008). Ding et al., 2021 observed decreased Mast1 expression in gastric tissue irradiated mice. Through genetic manipulation experiments, they revealed that Mast1 upregulation inhibits p38 MAPK signaling pathway activation, thereby alleviating radiation-induced gastric damage in mice. Our experimental results reveal that PVP-Se NPs decreased the expression of p-IκB-α and p-p65 in the NF-κB signaling pathway, as well as p-JNK, p-ERK, and p-p38 in the MAPK signaling pathway. Consequently, we hypothesize that PVP-Se NPs might be involved in radiation protection by reducing oxidative stress and inflammatory factors by inhibiting NF-κB and MAPK pathways.

## Conclusion

In summary, PVP-Se NPs can resist X-ray induced HUVECs cell damage and rat radiation damage, and have radiation protective effects both *in vivo* and *in vitro*. The mechanism may be related to reducing oxidative stress, inhibiting inflammatory factor expression, and regulating NF-κB and MAPK signaling pathway gene expression. We believe that future research should prioritize several key areas. Firstly, there is a need to optimize the synthesis methods of nanomaterials to enhance the stability and biocompatibility of PVP-Se NPs *in vivo*, while also minimizing potential toxicity and side effects. Secondly, clinical trials should be conducted to assess the radiation protection efficacy of PVP-Se NPs in humans. Thirdly, exploration of the potential application of PVP-Se NPs in protecting against radiation contamination could help safeguard both humans and ecosystems from radiation hazards. However, it's important to acknowledge the limitations of this study. Firstly, the investigation of the radiation protective effects of PVP-Se NPs *in vitro* was limited to only 1 cell line (HUVECs). Secondly, the evaluation of PVP-Se NPs’ radiation protection effects *in vivo* was restricted to the liver and kidney of rats. Thirdly, the analysis was focused solely on the expression of NF-κB and MAPK signaling pathway-related proteins in HUVECs and rats, with no rescued experiments conducted.

## Data Availability

The original contributions presented in the study are included in the article/Supplementary material, further inquiries can be directed to the corresponding author.
